# Cardiovascular health research priorities in the United Arab Emirates

**DOI:** 10.3389/fpubh.2023.1130716

**Published:** 2023-03-09

**Authors:** Nariman Ghader, Nabeel Al-Yateem, Sarah Dalibalta, Hira Abdul Razzak, Syed Azizur Rahman, Fatima Al Matrooshi, Sara Al Shaya, Amina Al Marzouqi

**Affiliations:** ^1^Department of Mental Health, Expert-Strategic Planning and Institutional Performance Management, Emirates Health Services, Dubai, United Arab Emirates; ^2^College of Health Sciences, University of Sharjah, Sharjah, United Arab Emirates; ^3^Department of Biology, Chemistry, and Environmental Sciences, American University of Sharjah, Sharjah, United Arab Emirates; ^4^Statistics and Research Centre, Ministry of Health and Prevention, Dubai, United Arab Emirates; ^5^Emirates Health Services, Dubai, United Arab Emirates

**Keywords:** cardiovascular diseases, cardiovascular health, research priorities, nominal group technique, United Arab Emirates

## Abstract

**Background:**

Cardiovascular diseases (CVDs) are a leading cause of morbidity and mortality in the United Arab Emirates (UAE) and have been prioritized for intervention by healthcare authorities and clinicians.

**Aim:**

To identify clinically relevant research priorities for the treatment and prevention of CVDs in the UAE.

**Methods:**

This study used the nominal group technique to identify CVD-related research priorities. Participants were 37 experts from UAE hospitals, academic and research institutions, CVD associations, and paramedical organizations.

**Results:**

Initially, 138 research topics were suggested by participating experts. These topics were then refined to identify the most important research priorities related to CVD prevention and treatment. The top research priority areas were: development of evidence-based, customized algorithms for CVD prevention and in-hospital emergency interventions; the availability, accessibility, and affordability of CVD treatment and rehabilitation; identification of relationships between CVDs, lifestyle factors, and mental health; efficacy and constraints in the management of cardiac emergencies; and epidemiological studies that trace CVD in the UAE.

**Conclusion:**

The identified research priorities will guide a more informed research program for CVD treatment and prevention in the UAE. Funding opportunities and support for researchers should be prioritized for these identified research areas.

## Introduction

Cardiovascular diseases (CVDs) are a leading cause of death globally (1) and account for 40% of mortality in the United Arab Emirates (UAE) (2, 3). The recent UAE National Health Survey identified CVDs as a significant health burden in the UAE, with physical inactivity, hypertension, obesity, and tobacco use being the top risk factors for CVD-related death (4). The UAE Health Vision 2021 focused on addressing CVD risk factors and decreasing mortality (5). Therefore, CVD-related research has been allocated a large amount of funding.

Given global and local statistics related to CVDs, the UAE developed a national strategic plan to reduce CVD-related mortality with input from local stakeholders. The unpublished document lists 52 initiatives that predominantly revolve around community outreach; this approach aimed to empower the public as a partner in assuming national responsibilities and act as a catalyst to introduce positive health changes to people's lifestyles. However, the government's directives to achieve the ultimate goal of reducing CVD-related mortality and the content of relevant policies and strategic plans still need to be backed up by empirical research. The existence of solid research will guide implementation of the strategy and provide a scientific platform for measuring outcomes and revising plans and interventions (6). The need for clinically relevant and focused healthcare research that contributes to improved healthcare services has also been recognized internationally (7). Many international health organizations have worked toward identifying research priorities to inform strategic plans for various disciplines. In 2011, the WHO issued a prioritized research agenda for the prevention and control of non-communicable diseases. This agenda stressed prevention and quality care, which required continuous input from updated research work to generate additional evidence-based knowledge and fill chasms in certain emerging areas (8).

The UAE Ministry of Health and Prevention (MOHAP) implemented an initiative to identify CVD-related research priorities to support the fight against CVDs and help the country update its CVD research agenda consistent with international trends. The majority of health policies and guidelines in the UAE are aligned with major global initiatives, such as the United Nations Sustainable Development Goals (9) and WHO research directives, and the perspectives of professional health organizations such as the World Heart Federation. However, in addition to keeping up with international efforts, it is important to incorporate local needs and factors specific to the UAE population and culture. A strong UAE research program will support a structured and evidence-based approach to respond to this global and national health burden, and help in identifying health research priorities for funding allocation. It will also help prioritize efforts to respond to the most relevant and urgent professional, public, and national needs in relation to CVDs (10) and update current research to match international efforts while considering local needs. The aim of this study, therefore, was to identify clinically relevant research priorities for the treatment and prevention of CVDs in the UAE.

## Methods

### Study design

The nominal group technique (NGT) was used to achieve the objectives of this study. NGT is a structured group discussion method which allows a set of priorities for action to be developed by consensus. NGT is particularly suited to learning about healthcare problems and can generate important solutions, thereby helping to bridge the gap between research and policymaking (11, 12).

Traditionally, NGT gathers group members physically in one place for a group meeting. However, given restrictions related to the COVID-19 pandemic and resulting increased use and acceptance of remote meeting technologies, it was considered appropriate to replace the physical meeting with online techniques. Therefore, we used a modified NGT process with online meetings and voting technologies. As well as being cost effective, this approach was practical as it allowed the inclusion of experts from diverse settings regardless of distance. Participating in this study was easy for these experts as they did not have to leave their workplace and travel to participate in the meeting. This enhanced attendance, thereby enriching the discussion and improving the study's outcomes.

### Participant recruitment

This study used expert sampling technique to recruit participants; this was consistent with the NGT method, where experts in the relevant area of research are selected to participate and give their expert input. The research team from MOHAP, the leading institution in this study, sent invitations through the research department and proper administrative channels to all local and federal government healthcare institutions, cardiovascular health non-government organizations, research institutions, and universities to nominate CVD experts to participate in the NGT meeting. It was specified in the nomination request that all nominated experts must have worked in the cardiovascular specialty area for at least 5 years in the UAE and have been based in the UAE for an uninterrupted period of at least 5 years.

In total, 33 CVD experts were nominated as representatives from different institutions under the authority of the MOHAP, Department of Health-Abu Dhabi, Abu Dhabi Health Services, Dubai Health Authority, Abu Dhabi Police, Dubai Corporation for Ambulance Services, Emirates Cardiac Society, National Ambulance, American Heart Association, the University of Sharjah, and the American University of Sharjah. In addition, five lay people from participating institutions were invited to represent the public's views. Selected experts from participating institutions received e-invites to participate in the event. The date and time for the meeting were coordinated, and all participants were provided with a link to join the online teleconference from their facility.

### Data collection procedure

#### Pre-workshop procedure

The research team held multiple meetings to design the workshop content and logistics. Upon completion of the workshop content, the team conducted a pilot meeting with five invited professionals that were not among the participating institutions' representatives. This offered opportunity for the research team to practice the workshop management process. The interaction of and feedback from pilot participants as well as the overall observations of the researchers were used to refine the workshop plan. Next, the team sent out e-invites with pre-reading materials to all nominated representatives, along with instructions for how to access the online meeting room. Microsoft Teams software was used to conduct the online meetings.

#### NGT meeting

The NGT meetings took place in October 2020. In total, 37 attendees and three moderators joined the meeting over 2 days. During the opening session on the first day, the moderator presented the workshop's goals and explained the corresponding exercises. Next, as per the NGT process, the moderator asked participants to complete a 5-minute individual thinking exercise (silent idea generation), after which they were asked to privately post their ideas (bullet points) using an online survey platform (i.e., online round-robin recording of ideas). The silent ideas generation and round-robin recording of ideas phases generated 216 ideas for research topics related to CVD prevention and treatment. In exercise two, which lasted 45 min, each participant was invited to discuss their ideas before all attendees in an idea clarifying or discussion phase. During this discussion, these ideas were processed by research assistants, and repeated or similar ideas were merged. The refining resulted in a list of 138 research ideas related to CVD prevention and treatment. These ideas were then put forward for final ranking and prioritizing by all participants as per the NGT protocol. This step was also performed using the online survey system. All ideas were presented to participants, and they were asked to select the five most important ideas. This ranking resulted in the selection of 29 ideas considered the most important, 27 ideas considered the second most important, 29 ideas considered the third most important, 29 ideas considered the fourth most important, and 31 ranked as the least important.

An additional round of ranking was undertaken to further refine these research priorities and select the most important research areas for CVD-related research in the UAE. This round aimed to identify the top research priorities in treating and preventing CVDs in the UAE. The 29 most important ideas were organized in an electronic survey and posted to participants. Participants were asked to rank this list of research priorities one more time for its importance and select the 5 most important priorities.

### Data analysis

A strength of the NGT approach is that results are produced instantly during the meeting as participants vote and revote on generated ideas. This allowed all workshop participants to contribute to generating a list of research priority areas for CVD prevention and treatment. Data analysis in NGT starts as the group meeting starts, and participants generated ideas are recorded. Within this study, this process was done electronically. The ideas were then placed in front of the group members for a thorough discussion; similar ideas were merged during these discussions. Finally, when the final list of ideas was generated, rounds of voting took place until the majority agreed on the top priority research areas. During the final round of voting, participants were asked to rank each identified priority on a scale from 1-10; the mean score of importance for participants' votes was then calculated for each priority area (i.e., the total sum of ratings divided on the total number of participants).

### Ethics

This study was approved by the MOHAP Research Ethics Committee (approval reference no. MOHAP/DXB-REC/AAA/No.40/2020). The research team ensured that all participants had read the study information sheet and provided signed written informed consent. The consent form was emailed to the nominated participants by email. Participants were asked to sign the consent and return back before the meeting time. All data were handled in accordance with the MOHAP data privacy and protection policy and the UAE data and privacy protection law (13) All data obtained from participants were kept confidential.

## Results

### Participants

In total, 37 participants took part in the two modified NGT meeting sessions. Participants included consultant cardiologists, representatives from the ambulance services and emergency medicine, family medicine, a neurologist, representatives from health services management, representatives from local and international CVD-related associations, and a group of academics, researchers, and community members active in the area of CVD prevention, treatment, and research.

### Priority themes

The identified CVD research priorities were: (1) development of customized algorithms for CVD prevention and in-hospital emergency interventions; (2) availability, accessibility, and affordability of CVD treatment and rehabilitation; (3) relationship between CVD, lifestyle, and mental health; (4) efficacy of and constraints on medical services in the management of cardiac emergencies; and 5) epidemiological studies in the area of CVDs in the UAE. Table 1 summarizes the broad priority areas and their mean score of importance.

**Table 1:** Research priority areas and their mean score of importance.

	**Priority**	**Mean score of importance**
1.	Development of customized algorithms for CVD prevention and in-hospital emergency interventions	8.34
2.	Availability, accessibility, and affordability of CVD treatment and rehabilitation	8.21
3.	Relationship between CVD, life style, and mental health	7.95
4.	Efficacy and constraints to medical services in the management of cardiac emergencies	7.95
5.	Epidemiological studies	7.1
6.	Efficacy and barriers to CVD awareness programs	7.13
7.	Studies on public involvement in fighting against CVD	7.13
8.	Guidelines for CVD diagnosis and treatment in women and children	7.08
9.	The genetic and epigenetic links to CVD	6.47

## Discussion

This study identified top-priority research areas as a first step toward tackling CVDs as a national health crisis. The next step should be to design and implement national interventions and strategies based on these priority areas. Five research priority areas were identified that cover CVDs management and prevention. The identified research priorities include the development of customized algorithms for CVD prevention and in-hospital emergency interventions; researching the availability, accessibility, and affordability of CVD treatment and rehabilitation; researching the relationship between CVD, lifestyle, and mental health within the UAE context; researching the efficacy of and constraints on medical services in the management of cardiac emergencies; and finally conducting epidemiological studies in the area of CVDs in the UAE.

### Research area 1: Development of customized algorithms for CVD prevention and in-hospital emergency interventions

Methods of predicting who may develop CVD are challenging, and several algorithms have been developed for population-based estimation of CVD risk. These include the Framingham risk score, Pan-European score, Reynolds risk score, ASSIGN Scottish algorithm, and the QRISK2 UK algorithm (14) A recent UAE study investigated some CVD risk assessment tools in a group of 2,621 participants with no history of CVD (15) Those authors found low-to-moderate overall agreement between the different risk assessment tools, which highlighted the need to improve existing tools or generate new tools based on data from the region. In addition, most current algorithms were developed for population-based predictions, which differ from personalized forecasts. Therefore, novel strategies such as high-throughput derived omics biomarkers and genome-scale metabolic models may be better suited for personalized treatment (16) Moreover, algorithms can significantly enhance the efficacy of hospital emergency systems and accurately predict the severity of a patient's medical condition. Machine-learning algorithms, such as logistic regression, Bayesian networks, and deep learning, have been deployed in medicine to predict patient admission and improve patient triage with relatively high accuracy (70–90%) (17).

### Research area 2: Investigating the availability, accessibility, and affordability of CVD treatment and rehabilitation

There is a paucity of research discussing the accessibility and affordability of CVD treatment in the UAE. However, evidence suggests that total expenditure on health has increased, and that progress has been made in the healthcare system with generally high patient satisfaction. The UAE has state-of-the-art facilities, and spends an estimated 13.6 billion USD on healthcare, although there are variations in access, affordability, and quality across the Emirates (18) The UAE also aimed to implement extensive health system reforms consistent with the country's 2021 vision that all Emiratis and residents have access to comprehensive, world-class facilities for early diagnosis and preventive medicine. Further research in the UAE should investigate the costs and accessibility of CVD treatment and the promotion of resources to support more cost-effective strategies, such as CR.

### Research area 3: Relationship between CVD, lifestyle, and mental health

There is an established relationship between poor lifestyle choices and CVD. These lifestyle factors include bad dietary habits, physical inactivity, adiposity and dyslipidemia, excessive stress, hypertension, diabetes mellitus, and smoking (19) These factors have therefore become targets for the assessment of CVD risk and treatment and monitoring of patients with CVD. Notably, modest adjustments of these lifestyle risk factors can have substantial improvements on cardiovascular risk. In the UAE, these risk factors are prevalent, as the country has some of the highest rates of physical inactivity globally(20) with nearly 58% of the adult population being physically inactive (4). The prevalence of overweight/obesity is approximately 71% in UAE adults (6) and over 30% in children (21, 22) Statistics also suggest the UAE has the second highest prevalence of diabetes globally (18.7% of the population are affected) (20) and smoking is also common (9.1% of the population are smokers) (23). This further emphasizes the need to allocate more resources to researching lifestyle risk factors for CVD in the UAE, raising population awareness, and establishing effective interventions.

It is also important to note the influence of mental health on CVD risk. Mental distress (e.g., depression and anxiety) can increase the risk for developing or worsening CVD conditions by about 80% (24) It can also contribute to increased blood glucose levels, weight gain, unhealthy lifestyles, and increased blood pressure. Mental health disorders are prevalent in the UAE, with a cross sectional study revealing 57.2% of surveyed participants had suffered from at least one mental disorder, with higher rates in women(25, 26). The most common disorders were anxiety (56.4%), depression (31.5%), posttraumatic stress disorder (15.1%), and phobic disorders (10.8%). These findings also highlight the need for interventions in investigating mental health disorders in this population, another risk factor for CVD, and the necessity to improve mental health outcomes.

### Research area 4: Understanding the efficacy of and constraints on medical services in the management of cardiac emergencies

Cardiac emergencies are a leading cause of death. Therefore, it is vital to establish proper clinical practice guidelines for the initial evaluation and treatment of patients with symptoms of a cardiac emergency both pre-hospital and in-hospital. There have been major advances worldwide in cardiopulmonary resuscitation (CPR) and defibrillation, which are known to significantly increase survival rates from cardiac arrest when performed early. The UAE National Ambulance Service evaluated the characteristics of out-of-hospital cardiac arrest (OHCA) in a report published in 2019 (27) A total of 715 OHCA cases attended by National Ambulance crew were enrolled in that study. Although cardiac arrest was witnessed in more than half of these cases, only 53.2% of patients received bystander CPR. Moreover, an automated external defibrillator (AED) was only applied in two cases before the arrival of ambulance services. This highlighted a clear gap in the chain of survival with a number of barriers regarding OHCA, such as a lack of knowledge in recognizing cardiac arrest or a lack of confidence in performing CPR. Furthermore, the UAE does not have a Good Samaritan law and bystanders may fear legal action following their interference. There also appears to be low public access to AED. That study highlighted the need to improve public awareness of the symptoms of cardiac arrest, develop training programs on how to perform CPR, and enhance community engagement for better prognosis and survival.

### Research area 5: Performing epidemiological studies pertaining to CVD in this population

To fully understand the scale of the CVD issue in the UAE, it is imperative to collect current epidemiological data on CVD and its risk factors in this population, and implement strategies aimed at reducing its burden. The prevalence of CVD in the UAE is increasing, and it is necessary to target modifiable risk factors such as high cholesterol levels, obesity, physical inactivity, high blood pressure, smoking, and high blood glucose levels in this population (28) In addition to the individual (lifestyle) risk factors, there are also societal risk factors (health systems, prevention and care, medical emergency services) related to CVD that should be addressed. The WHO, Centers for Disease Control and Prevention, American Heart Association, and other stakeholders have called for action to meet the challenges of CVD and outline ways to improve prevention and care (29) The most cost-effective and economically feasible policies with the highest likelihood of success are intervention strategies that initiate interventions at the population level (30). The lack of reliable data from the UAE population that accurately estimates the full burden of CVD hinders the establishment of nationwide prevention and management strategies. Therefore, there is a need for better health information and epidemiological studies to monitor progress and guide health policy decisions in CVD prevention and management using evidence-based and cost-effective preventive approaches.

## Conclusions and implications of our findings

By bringing together a group of multidisciplinary participants across the spectrum of health governing bodies in the UAE using a virtual platform, the MOHAP has been successful in creating a plan to transform the UAE's public health strategy for CVD prevention and intervention. The recommendations derived from this meeting represent a range of relevant perspectives and provide a framework for the development of national guidance on the prevention of CVD. The enormous burden of CVD in terms of suffering and healthcare costs is escalating, with a clear need for comprehensive action plans. The research themes that emerged from this should guide the research agenda in the country for the coming few years. Research funding should therefore be directed to the priority areas identified, which also can provide a basis for cooperation among research funders and key partners.

## Study limitations

This study has some limitations. First is the fact that this is a national-level study. The importance of the study is therefore limited to the UAE healthcare system context. However, the study could be a landmark for future similar regional studies.

The experts who participated in the study were from local institutions, experts from international organizations such as WHO were not included. The inclusion of international experts could have enriched the study results.

Finally, the generalizability of the study may be limited due to a potential selection bias. The participating experts were from specific institutions accessed and invited by the MOHAP, and from institutions that responded to this invitation. Therefore, the study findings could not be generalized beyond the experts who participated in this study.

## Data availability statement

The raw data supporting the conclusions of this article will be made available by the authors, without undue reservation.

## Ethics statement

The studies involving human participants were reviewed and approved by the MOHAP Research Ethics Committee (approval reference no. MOHAP/DXB-REC/AAA/No.40/2020). The patients/participants provided their written informed consent to participate in this study.

## Author contributions

All authors listed have made a substantial, direct, and intellectual contribution to the work and approved it for publication.
